# Association of living environmental and occupational factors with semen quality in chinese men: a cross-sectional study

**DOI:** 10.1038/s41598-023-42927-z

**Published:** 2023-09-21

**Authors:** Hanran Mai, Junyi Ke, Miaomiao Li, Menghua He, Yanxia Qu, Fan Jiang, Simian Cai, Yufen Xu, Lanyan Fu, Lei Pi, Huazhong Zhou, Hongyan Yu, Di Che, Xiaoqiong Gu, Jinxin Zhang, Liandong Zuo

**Affiliations:** 1 Department of Clinical Biological Resource Bank, Guangzhou Institute of Pediatrics, Guangzhou Women and Children’s Medical Center, Guangzhou Medical University, Guangzhou, 510623 China; 2 Department of Andrology, Guangzhou Women and Children’s Medical Center, Guangzhou Medical University, 9 Jinsui Road, Guangzhou, 510623 Guangdong China; 3 Department of Medical Statistics, Sun Yat-Sen University, Guangzhou, 510080 China; 4 Department of Laboratory, The Second Affiliated Hospital of Guangzhou Medical University, Guangzhou, 510260 China; 5 Department of Gynecology, Guangzhou Women and Children’s Medical Center, Guangzhou Medical University, Guangzhou, 510623 China; 6 Prenatal Diagnostic Center, Guangzhou Women and Children’s Medical Center, Guangzhou, 510623 Guangdong China; 7 Department of Science, Education and Data Management, Guangzhou Women and Children’s Medical Center, Guangzhou, 510623 Guangdong China

**Keywords:** Medical research, Risk factors, Urology

## Abstract

Sperm quality can be easily influenced by living environmental and occupational factors. This study aimed to discover potential semen quality related living environmental and occupational factors, expand knowledge of risk factors for semen quality, strengthen men's awareness of protecting their own fertility and assist the clinicians to judge the patient’s fertility. 465 men without obese or underweight (18.5 < BMI < 28.5 kg/m^2^), long-term medical history and history of drug use, were recruited between June 2020 to July 2021, they are in reproductive age (25 < age < 45 years). We have collected their semen analysis results and clinical information. Logistic regression was applied to evaluate the association of semen quality with different factors. We found that living environment close to high voltage line (283.4 × 10^6^/ml vs 219.8 × 10^6^/ml, Cohen *d* = 0.116, P = 0.030) and substation (309.1 × 10^6^/ml vs 222.4 × 10^6^/ml, Cohen *d* = 0.085, P = 0.015) will influence sperm count. Experienced decoration in the past 6 months was a significant factor to sperm count (194.2 × 10^6^/ml vs 261.0 × 10^6^/ml, Cohen *d* = 0.120, P = 0.025). Living close to chemical plant will affect semen PH (7.5 vs 7.2, Cohen *d* = 0.181, P = 0.001). Domicile close to a power distribution room will affect progressive sperm motility (37.0% vs 34.0%, F = 4.773, Cohen *d* = 0.033, P = 0.030). Using computers will affect both progressive motility sperm (36.0% vs 28.1%, t = 2.762, Cohen *d* = 0.033, P = 0.006) and sperm total motility (57.0% vs 41.0%, Cohen *d* = 0.178, P = 0.009). After adjust for potential confounding factors (age and BMI), our regression model reveals that living close to high voltage line is a risk factor for sperm concentration (Adjusted OR 4.03, 95% CI 1.15–14.18, R^2^ = 0.048, P = 0.030), living close to Chemical plants is a protective factor for sperm concentration (Adjusted OR 0.15, 95% CI 0.05–0.46, R^2^ = 0.048, P = 0.001) and total sperm count (Adjusted OR 0.36, 95% CI 0.13–0.99, R^2^ = 0.026, P = 0.049). Time spends on computer will affect sperm total motility (Adjusted OR 2.29, 95% CI 1.11–4.73, R^2^ = 0.041, P = 0.025). Sum up, our results suggested that computer using, living and working surroundings (voltage line, substation and chemical plants, transformer room), and housing decoration may association with low semen quality. Suggesting that some easily ignored factors may affect male reproductive ability. Couples trying to become pregnant should try to avoid exposure to associated risk factors. The specific mechanism of risk factors affecting male reproductive ability remains to be elucidated.

## Introduction

As the problem of astogeny and birth rates falling became more popular worldwide, infertility has significantly negative affecting on overall fertility and family harmony^1^. Infertility is a disease that defined as fail to conceive after 12 months of regular and unprotected sexual intercourse. In recent decades, affect by multiple negative factors, the infertility rate increased significantly worldwide, which is about 12–20%^2,3^. The infertility rate in China is about 12.5%, and is also rising. In up to 40% of the infertility couples, men must be responsible for the inability to conceive^4^.

Male infertility is related with a few varieties of causation. Except for the irreversible reasons (such as genetic), and the organic diseases like varicocele, we should also pay attention to demographic factors. For example, occupations and living environment, which may not be so influential as organic disorder and genopathy to male fertility, yet these factors are easy to be ignored. But for most of people, they tend to maintain only one or several similar occupations and seldom change their living environment, this may lead to the potential infertility related factors of environment and occupations influence people’s fertility for their entire life. Furthermore, manifold factors can affect together as an additive effect, may result in an infertility phenotype even more serious than organic disorder. Thus, in addition to clinical diagnosis or medical research on male infertility, attention also should be paid on the influence from occupational and environmental factors on male fertility.

A mass of researches have been conducted about effect of occupational and environmental factors on male fecundity, studies about occupations and semen quality indicated that sperm of the people engaged in the transportation business have the lowest motility^5,6^. Another research had pointed out that occupations like farmer, workers in printing factories and oil workers who are close to toxic chemicals are related to poor male fertility^7,8^. Although many researches are supporting the view that occupations are relevant to semen quality, some papers have different standpoint, they drew a conclusion that occupation had no significant association to semen quality^9^. This kind of discrepancy may due to variety of occupations and population differences and more explorations are needed.

Living environmental factors are also related to male fertility and plenty of relevant researches had been conducted. A meta-analysis conducted by J. A. Adams had shown that cellular telephones using may negatively correlate with sperm motility but have no relationship with sperm concentration^10^. Abdollahi held a single fertility center cohort study which indicated that environmental noise will result in the low motility and abnormality of sperm^11,12^. Houses decoration also potentially affect male fertility. During the decoration, there are mainly three toxic substances correlated with semen quality: benzene, formaldehyde and ammonia. And these kinds of toxins remain high levels of concentration in the house after the decoration. Researches had shown that these substances are highly relevant to male infertility^13,14^.

To learn as much as we can about the environmental and occupational factors our patients have experienced. We had designed three questionnaires about demographic characteristics and living environmental and occupational factors as exposures. We constructed these questionnaires base on the living habits of most Chinese people.

Herein, based on our fertility cohort, more than 465 couples were enrolled to this research during June 2020 to July 2021. We had collected couples’ essential information as well as occupation and environment expose questionnaires. In addition, we had finished these couples’ pregnancy follow-up visits. This study intends to explore which occupational and environmental factors related to low semen quality and influence the likelihood of a successful pregnancy.

## Materials and methods

### Study population

We enrolled couples from Guangzhou Women and Children's Medical Center in China, Guangzhou for free pre-pregnancy medical examinations. As a national welfare of China, this program, provided as part of China's national welfare system, allows couples to undergo comprehensive physical check-ups before marriage and planning pregnancy to ensure the health of both parents and the baby. They were invited to take part in a prospective cohort which were focused on the issue if occupational and environmental factors influence fertility. Herein, after excluded male partners with a medical history of systemic diseases, infertility related disease (including varicocele, cryptorchidism, and azoospermia, etc.), obese or underweight (18.5 < BMI < 28.5 kg/m^2^), and long-term medication history, totally 465 male partners of couple age 31 to 43 years were included in this study between June 2020 to July 2021. All of them have completed three questionnaires which were about living environment, occupation, and basis information of demographic, respectively. The study population consisted of individuals of East Asian descent.

After excluding male partners with a medical history of systemic diseases, infertility-related diseases (including varicocele, cryptorchidism, and azoospermia, etc.), and long-term medication history, a total of 465 couples were included in this study between June 2020 to July 2021.

### Physical examination and semen analysis

Physical examinations and semen analyses were conducted on the same day. Participants' body mass index (BMI: weight divided by height squared (kg/m^2^)) was recorded, and the testicles and scrotums were examined to exclude individuals with varicocele or other abnormalities of the reproductive organs.

Participants were instructed to abstain from sexual activity for three to seven days before the semen analysis and physical examination. Semen samples were collected in a sterile semen container by masturbation and placed in a 37 °C incubator for 30 min to liquefy. After the liquefaction, semen analysis was performed by computer aid sperm analysis (CASA, SuiJia Software, Beijing, China) to evaluate semen PH, Semen volume, sperm concentration, sperm count, sperm progressive motility, total motility. All our operations and reference values of semen parameters followed the newest guidelines of the World Health Organization (WHO)^15^.

Our laboratory conducted quality control regularly to guarantee the high quality of the semen analysis results.

### Environment and occupation questionnaires

According to the living and working habits of people in China, we design two separate questionnaires to access participants’ environmental and occupational exposures. The questionnaires included items related to previously reported factors associated with low semen quality, such as painters^7,16^, drivers^17^, and office staff^18,19^. We had also designed a few extra questions for the basis demographic characteristics. Our questionnaires would be performed as multiple-choice questions.

Designed by experts from the Department of pre-marriage and pre-pregnancy health care of Guangzhou Women and Children Medical Center, the first pilot test was carried out in Wanqingsha Hospital, Nansha District, Guangdong Province. Finally, part of the questionnaires was modified according to the pilot testing results, and then conduct this study.

### Ethics statement

The present study protocol was reviewed and approved by the Ethics Review Committee of the Guangzhou Women and Children's Medical Center (2016102416). All procedures followed were in accordance with the ethical standards of the responsible committee on human experimentation (institutional and national) and with the Helsinki Declaration of 1975, as revised in 2000. Informed consent was obtained from all patients for being included in the study.

### Statistical analysis

Shapiro–Wilk test and histograms was applied to assessed the normality of the data. All the seminal parameters did not conform to the normality except progressive motility (%). All data was presented as median (25th, 75th percentiles). The association between semen quality parameters and environmental and occupational factors were evaluated, Mann–Whitney U-test and Kruskal–Wallis H test for the data with a non-normal distribution (pH value, semen volume, sperm concentration, sperm count, total motility) and ANOVA for the normally distributed data (progressive motility). In order to explain the practical value of the results and judge the impact of the sample size on the results of this study, we introduced Cohen's *d* value to represent the effect size^20^.

To further explore the association between semen quality and environmental and occupational factors. Binomial logistic regression was applied to detect the independent predictors which were significantly affect semen quality, confounders were adjusted for the analysis: education^21^, BMI^22^, smoking^23^, alcohol consuming^24^ and age^25^. We calculated the effect size of each factor using the Cohen *d* statistic for the two groups comparison, and eta-squared for the observations more than two sets^20,26^. All P-value of less than 0.05 was taken to indicate statistical significance. Statistical analyses were performed by using SPSS version 26.0 (SPSS Inc., Chicago, IL, USA).

### Preprint

A previous version of this manuscript was published as a preprint^27^.

## Results

### Characteristics of study population

As shown in Table 1, there were totally 465 males of reproductive age enrolled in this study, the mean age was 37.5 years (± 5.7 years) and the mean BMI was 23.85 kg/m^2^ (± 4.42 kg/m^2^). All participants had a permanent job and was willing to accept our follow-up service. Approximately 20.9% and 8.8% of our population are current alcohol consumers and smokers, respectively. Our study had included people of every degree of education.

**Table 1:** General characteristics of the study population (n = 465).

Variables	N (%) or Mean ± SD
Age, years	37.5 ± 5.7
Education, n (%)	
Primary school and below	11 (2.4)
Junior high school	90 (19.4)
High school	140 (30.1)
College or university degree	215 (46.2)
A master's degree or higher	7 (1.5)
N/A	2 (0.4)
BMI, kg/m^2^	23.85 ± 4.42
Alcohol consumers	
Yes, n (%)	97 (20.9)
No, n (%)	368 (79.1)
Smoker	
Yes, n (%)	41 (8.8)
No, n (%)	424 (91.2)

### Semen quality

According to our current study, the median (25th, 75th percentiles) values for semen PH was 7.4 (7.2–7.6), semen volume was 4.2 (2.6–5.2) ml, sperm concentration was 80.5 (37.0–103.6) × 10^6^/ml, sperm count was 341.6 (121.6–429.4) × 10^6^/ml, total motility was 54.3 (39.0–69.5) %, and the sperm progressive motility was 36.0 (22.0–48.0) (Table 2).

**Table 2:** Summary of semen parameters of males.

Variables	Statistics
pH value, Median (25th, 75th percentiles)	7.4 (7.2–7.6)
Semen volume(ml), Median (25th, 75th percentiles)	4.2 (2.6–5.2)
Sperm concentration (10^6^/ml), Median (25th, 75th percentiles)	80.5 (37.0–103.6)
Sperm count (10^6^), Median (25th, 75th percentiles)	341.6 (121.6–429.4)
Sperm progressive motility (%), (25th, 75th percentiles)	36.0 (22.0–48.0)
Total motility (%), Median (25th, 75th percentiles)	54.3 (39.0–69.5)

### Correlation between environment and occupation factors and semen quality

All semen parameters did not follow the normal distribution except progressive motility (%). Mann–Whitney U-test and Kruskal–Wallis H test were applied for analysis to semen measurements with non-normal distribution. ANOVA was applied for analysis to semen measurements with normal distribution. Our results suggested that male who lived within two kilometers of a high voltage line which is defined as distribution line AC voltage in more than 1000 voltage or DC voltage in more than 1500 V electrical connection line (283.4 × 10^6^ vs. 219.8 × 10^6^; *P* = 0.030; Cohen *d* = 0.116) or a substation (309.1 × 10^6^ vs. 222.4 × 10^6^; *P* = 0.015; Cohen *d* = 0.085) would increase the sperm count (10^6^/ml). However, when there were power distribution room located within two kilometers from our participants’ residences, their sperm progressive motility (%) decreased significantly (37.0% vs. 34.0%; F = 4.773, *P* = 0.030; Cohen *d* = 0.033). Living close to a chemical factory was another factor affecting semen quality, but based on our data from this research, although the semen PH was increased significantly (7.5 vs. 7.2; t = 2.762; *P* = 0.001; Cohen *d* = 0.181), but according to WHO’s guideline, the reference range for PH value is between 7.2 and 7.8. Therefore, whether living close to a chemical factory is a negative factor to human semen quality, more researches are needed. Decoration materials’ reproduction toxicity has got a lot of attentions. Our research found out that if anyone lives in a house undergone decoration within a half year, his sperm count would decrease (194.2 × 10^6^ vs. 261.0 × 10^6^; *P* = 0.025; Cohen *d* = 0.120). Another factor which has drawn much attention in recent years is computers using. We observed a decline of sperm progressive motility (within eight hours: 36.0%vs. more than 8 h: 28.1%; *P* = 0.006; Cohen *d* = 0.033) and sperm total motility (within 8 h: 57.0% vs. more than 8 h: 41.0%; *P* = 0.009; Cohen *d* = 0.178) in our participants who attach to computers every day (Table 3).

**Table 3:** Description of semen parameters in different residential environments and occupational exposures.

Characteristic	N	pH value		Semen volume(ml)		Sperm concentration(10^6^/ml)		Sperm count (10^6^)		Total motility (%)		Progressive motility (%)	
		Median (25th, 75th)	Effect size(d_s_)	Median (25th, 75th)	Effect size(d_s_)	Median (25th, 75th)	Effect size(d_s_)	Median (25th, 75th)	Effect size(d_s_)	Median (25th, 75th)	Effect size(d_s_)	Median (25th, 75th)	Effect size(d_s_)
High voltage line (within 2 km)													
Yes	207	7.5 (7.2–7.6)	0.023	4.1 (3.0–5.6)	0.098	64.9 (37.9–103.7)	0.039	283.4 (140.4–492.0) *	0.116	55.0 (39.0–70.8)	0.003	36.0 (22.5–50.0)	0.026
No	258	7.5 (7.2–7.6)		4.0 (2.5–5.3)		63.6 (38.2–107.8)		219.8 (102.2–435.4)		55.0 (41.0–69.0)		34.1 (22.0–47.0)	
Large substation (within 2 km)													
Yes	124	7.5 (7.2–7.5)	0.030	4.1 (3.0–6.0)	0.045	66.1 (38.3–108.7)	0.080	309.1 (145.3–511.0) *	0.085	57.0 (39.0–71.0)	0.047	36.0 (22.0–51.0)	0.037
No	341	7.5 (7.2–7.6)		4.0 (2.5–5.4)		63.6 (37.4–103.6)		222.4 (119.9–423.4)		55.0 (40.3–68.0)		35.0 (22.0–47.0)	
Power distribution room (within 2 km)													
Yes	231	7.5 (7.2–7.6)	0.043	4.1 (3.0–5.6)	0.043	62.2 (33.1–100.0)	0.032	256.4 (127.8–497.8)	0.037	55.0 (40.3–71.0)	0.030	37.0 (24.0–50.0) *	0.033
No	234	7.4 (7.2–7.6)		3.9 (2.5–5.4)		64.2 (41.0–108.2)		233.6 (121.8–419.4)		55.0 (37.5–68.0)		34.0 (19.5–47.0)	
A radio and television transmission tower (within 2 km)													
Yes	59	7.5 (7.2–7.7)	0.028	3.5 (2.5–6.0)	0.030	60.8 (24.7–93.5)	0.050	234.7 (97.9–537.2)	0.018	58.0 (41.0–74.0)	0.082	36.0 (21.0–53.3)	0.024
No	406	7.5 (7.2–7.6)		4.0 (3.0–5.5)		64.2 (40.3–108.0)		254.4 (127.8–457.6)		55.0 (39.5–69.0)		36.0 (22.0–47.9)	
Cell phone base station (within 2 km)													
Yes	138	7.4 (7.2–7.5)	0.106	4.0 (2.4–5.0)	0.082	53.7 (29.2–99.8)	0.044	228.9 (99.3–455.9)	0.029	55.0 (41.0–68.0)	0.015	36.8 (22.0–47.9)	0.031
No	327	7.5 (7.2–7.6)		4.0 (3.0–5.5)		65.6 (40.0–108.0)		254.4 (132.7–459.6)		55.0 (36.0–70.0)		34.0 (22.0–48.0)	
Chemical plant (within 2 km)													
Yes	71	7.5 (7.375–7.5)*	0.181	3.7 (2.8–6.0)	0.008	63.4 (35.5–126.0)	0.027	287.8 (104.4–563.0)	0.050	57.0 (41.8–72.0)	0.056	38.0 (21.0–52.0)	0.007
No	394	7.2 (7.2–7.4)		4.0 (2.8–5.4)		64.0 (38.0–103.6)		231.9 (127.6–442.0)		55.0 (39.0–69.0)		36.0 (22.0–48.0)	
Traffic artery (within 2 km)													
Yes	325	7.5 (7.2–7.6)	0.020	4.0 (2.9–5.4)	0.054	63.6 (35.0–100.9)	0.030	224.6 (120.6–463.7)	0.033	54.0 (37.8–69.0)	0.086	35.0 (22.0–47.9)	0.034
No	140	7.5 (7.2–7.7)		4.1 (2.8–5.5)		64.1 (43.2–116.9)		263.4 (145.4–437.4)		59.0 (45.0–72.3)		36.0 (21.0–48.0)	
Drinking water													
Tap water	372	7.5 (7.2–7.6)	0.087	4.0 (2.8–5.5)	0.490	64.2 (37.8–108.2)	0.091	262.3 (128.3–474.9)	0.178	57.0 (40.0–69.0)	0.236	34.2 (22.0–47.0)	0.032
Bottled water	64	7.5 (7.2–7.7)		3.8 (2.1–4.4)		65.1 (49.8–91.8)		206.0 (110.6–344.0)		52.0 (40.0–67.0)		41.0 (26.0–52.0)	
Spring water	6	7.6 (7.4–7.8)		4.6 (4.1–4.8)		25.1 (13.0–33.7)		90.3 (47.3–150.8)		39.0 (31.5–41.5)		23.6 (18.5–32.3)	
Other	23	7.4 (7.2–7.5)		5.0 (3.9–6.5)		56.5 (42.8–93.8)		338.8 (156.2–667.9)		59.0 (48.0–75.0)		43.0 (26.6–51.0)	
Buy a new car (within 6 months)													
Yes	54	7.5 (7.2–7.6)	0.044	3.8 (2.5–5.4)	0.011	78.9 (51.7–137.5)	0.084	261.0 (116.3–662.3)	0.058	55.0 (41.5–66.5)	0.003	33.5 (22.3–46.4)	0.019
No	411	7.5 (7.2–7.6)		4.0 (2.8–5.5)		61.3 (37.8–103.8)		237.7 (126.0–433.1)		55.0 (39.0–70.0)		36.0 (22.0–48.0)	
Decorate within half a year													
Yes	48	7.5 (7.2–7.8)	0.066	3.9 (3.0–4.8)	0.049	58.3 (24.0–96.2)	0.089	194.2 (77.0–351.1)	0.120	58.0 (33.5–70.0)	0.002	34.0 (26.3–51.8)	0.023
No	417	7.5 (7.2–7.6)		4.0 (2.8–5.5)		64.2 (38.7–108.0)		261.0 (130.7–478.1) *		55.0 (40.5–69.0)		36.0 (22.0–47.9)	
Purchase new furniture or painted furniture (within 6 months)													
Yes	70	7.5 (7.3–7.7)*	0.125	3.9 (2.2–5.0)	0.039	68.0 (29.2–102.1)	0.050	201.1 (83.1–463.1)	0.063	58.0 (38.5–70.0)	0.026	35.0 (28.0–51.0)	0.029
No	395	7.5 (7.2–7.6)		4.0 (2.9–5.5)		63.7 (37.9–108.1)		254.4 (128.3–442.4)		55.0 (40.0–69.0)		36.0 (21.8–47.9)	
Occupation													
Institutions, party organizations, enterprises, institutions	34	7.5 (7.2–7.6)	0.415	3.0 (2.0–5.0)	0.713	61.1 (30.6–84.6)	0.027	165.3 (99.3–333.8)	0.348	49.0 (35.0–68.0)	0.470	33.3 (13.8–45.7)	0.019
Professional skill worker	37	7.5 (7.2–7.6)		3.9 (2.1–5.0)		54.5 (28.3–98.8)		176.6 (108.6–425.8)		57.5 (45.3–72.5)		38.6 (31.1–50.0)	
Administrative, law enforcement, and clerical personnel	42	7.5 (7.2–7.5)		4.1 (3.0–5.5)		68.9 (46.8–121.3)		307.1 (157.9–551.4)		51.5 (39.5–66.0)		31.0 (20.8–46.1)	
Commercial and service industry personnel	56	7.5 (7.2–7.8)		4.1 (2.8–5.6)		80.0 (39.1–116.1)		296.4 (165.1–522.2)		58.0 (42.3–68.0)		34.1 (22.3–51.5)	
Production personnel in agriculture, forestry, animal husbandry, fishery and water conservancy	15	7.4 (7.2–7.5)		3.5 (2.2–4.6)		89.6 (36.0–151.8)		258.5 (115.4–448.6)		63.5 (28.0–84.0)		32.0 (20.0–44.0)	
Production and transportation equipment operators and related personnel	35	7.5 (7.2–7.5)		4.8 (3.3–7.6)		61.0 (32.3–95.6)		301.0 (125.2–525.6)		60.5 (51.8–68.5)		37.0 (24.0–45.0)	
Unemployment	32	7.3 (7.2–7.5)		4.1 (2.9–5.4)		57.1 (28.9–99.9)		203.8 (145.7–386.5)		55.0 (39.0–69.5)		34.0 (22.0–55.3)	
Retire	16	7.5 (7.3–7.7)		3.7 (3.0–4.8)		64.9 (48.2–85.8)		258.6 (146.6–424.1)		63.5 (41.3–77.3)		43.0 (19.8–51.8)	
Other	86	7.5 (7.2–7.6)		4.0 (3.0–5.6)		58.1 (37.9–99.3)		223.2 (115.5–493.8)		54.0 (34.0–69.0)		36.0 (22.0–48.0)	
Nature of work													
Chemical	11	7.6 (7.2–7.7)	0.339	3.1 (2.0–7.3)	0.236	69.3 (32.6–117.6)	0.250	316.1 (114.4–676.1)	0.215	53.5 (41.0–67.3)	0.470	40.0 (35.0–52.3)	0.026
Manufacturing	83	7.5 (7.2–7.7)		4.1 (3.0–6.0)		58.3 (32.3–93.4)		220.2 (125.2–374.2)		54.0 (36.5–68.3)		37.3 (25.5–45.5)	
Catering	24	7.5 (7.2–7.8)		4.4 (2.7–5.4)		93.7 (42.0–137.0)		368.5 (133.9–670.0)		61.0 (48.0–78.0)		36.0 (23.0–48.0)	
Transportation	13	7.5 (7.2–7.6)		4.2 (2.8–5.0)		70.4 (37.0–113.9)		295.6 (82.0–532.5)		56.0 (45.0–67.0)		31.5 (22.3–36.0)	
Environmental protection	4	7.4 (7.1–7.7)		4.8 (3.3–5.8)		70.0 (53.6–165.3)		267.9 (229.2–950.0)		64.5 (44.5–79.3)		35.5 (34.3–40.5)	
Medicine	26	7.5 (7.4–7.5)		5.0 (3.9–5.8)		68.0 (33.7–121.1)		333.8 (126.9–642.6)		70.0 (49.0–84.0)		37.0 (25.3–59.9)	
Farming	47	7.4 (7.2–7.5)		3.5 (2.5–4.5)		73.9 (35.9–105.4)		194.7 (107.2–479.9)		59.0 (32.5–80.5)		33.0 (22.0–51.5)	
Other	257	7.5 (7.2–7.6)		4.0 (2.8–5.5)		62.6 (37.8–107.7)		251.8 (119.3–442.4)		53.0 (36.0–68.0)		34.0 (20.0–48.5)	
Radioactive material contact													
Yes	15	7.5 (7.3–7.6)	0.050	4.8 (3.4–5.8)	0.060	68.0 (59.7–104.9)	0.095	342.3 (243.7–555.4)	0.095	67.0 (54.5–86.0)	0.316	48.0 (36.0–59.9)	0.025
No	369	7.5 (7.2–7.6)		4.0 (3.0–5.5)		62.5 (36.0–105.1)		248.9 (121.7–441.1)		55.5 (40.0–69.0)		35.0 (22.0–47.0)	
Unknown	81	7.4 (7.2–7.7)		3.9 (2.5–5.2)		67.4 (41.3–115.0)		233.6 (126.0–524.9)		51.0 (35.0–69.0)		38.0 (21.0–52.0)	
Toxic substances contact													
Yes	30	7.5 (7.2–7.6)	0.003	4.2 (2.3–5.8)	0.248	74.3 (42.2–170.2)	0.023	342.3 (137.2–681.0)	0.066	55.0 (47.5–77.0)	0.036	38.0 (32.0–44.0)	0.029
No	364	7.5 (7.2–7.6)		4.0 (3.0–5.5)		62.6 (37.9–101.8)		234.8 (124.3–432.1)		56.0 (39.0–69.0)		35.0 (22.0–48.0)	
Unknown	71	7.5 (7.2–7.7)		3.4 (2.1–5.0)		67.1 (34.7–123.3)		247.3 (120.8–523.1)		53.5 (35.5–72.0)		36.5 (18.5–52.0)	
Average daily mobile phone talk time (within 6 months)													
Less than 10 min	201	7.5 (7.2–7.6)	0.409	4.0 (2.5–5.0)	0.128	60.9 (35.7–99.3)	0.025	204.9 (106.2–392.8)	0.208	54.0 (39.0–68.0)	0.198	36.0 (22.0–47.0)	0.026
10–30 min	188	7.5 (7.2–7.6)		4.0 (2.8–5.4)		65.1 (40.9–115.1)		263.6 (128.3–442.4)		59.0 (42.0–72.0)		36.0 (23.0–48.0)	
30 ~ 60 min	40	7.4 (7.2–7.5)		4.6 (3.0–6.0)		80.4 (37.9–109.8)		276.0 (140.7–665.4)		49.5 (37.3–67.0)		33.5 (23.3–48.0)	
60 min and above	35	7.2 (7.2–7.5)		4.5 (3.4–8.5)		65.2 (39.1–110.5)		346.9 (201.7–613.7)		53.0 (28.5–69.0)		27.6 (11.3–44.8)	
Where to carry your phone													
Pockets near the waist	29	7.5 (7.2–7.8)	0.225	3.0 (2.0–5.0)	0.227	61.1 (40.9–162.8)	0.296	206.0 (122.2–366.7)	0.430	46.0 (33.0–68.0)	0.199	26.0 (14.5–40.4)	0.023
Hang on the chest or put it in a pocket near the chest	5	7.4 (7.0–.)		7.3 (7.0–.)		125.0 (61.3–.)		922.2 (429.4–.)		54.0 (42.0–.)		22.0 (16.7–.)	
Pants pocket	142	7.4 (7.2–7.5)		4.1 (2.8–6.0)		65.2 (41.3–130.9)		305.7 (126.3–560.2)		59.0 (41.0–71.0)		38.0 (22.0–50.0)	
Put in the bag	265	7.5 (7.2–7.6)		4.0 (2.8–5.0)		62.2 (32.5–99.9)		229.5 (114.7–374.8)		54.0 (39.8–69.3)		35.0 (22.0–47.2)	
Other locations	24	7.5 (7.2–7.7)		4.0 (3.0–6.6)		66.2 (51.2–88.7)		237.4 (182.0–547.2)		56.5 (39.8–63.8)		37.5 (33.0–51.8)	
Whether to shut down cellphone while sleeping													
Yes	46	7.5 (7.3–7.7)	0.092	3.8 (2.4–7.5)	0.016	61.7 (40.8–100.4)	0.033	209.3 (125.1–393.8)	0.048	55.0 (41.0–68.8)	0.002	36.0 (23.5–53.0)	0.037
No	419	7.5 (7.2–7.6)		4.0 (2.8–5.3)		64.1 (37.7–105.1)		253.1 (124.6–459.5)		55.0 (39.0–69.3)		36.0 (22.0–47.0)	
If it is not turned off, whether the phone is placed on the bed or placed within 1 m from the bed													
Yes	331	7.5 (7.2–7.6)	0.052	4.0 (2.8–5.4)	0.033	65.1 (37.9–115.1)	0.071	234.7 (126.2–463.1)	0.038	55.0 (39.0–68.0)	0.046	36.0 (22.8–48.0)	0.018
No	134	7.5 (7.2–7.6)		4.0 (2.8–5.5)		59.0 (37.8–90.3)		254.4 (117.1–431.2)		57.0 (41.0–73.0)		35.0 (21.0–45.0)	
Stop using mobile phone during planned pregnancy													
Have been using	422	7.5 (7.2–7.6)	0.020	4.0 (2.8–5.5)	0.052	64.0 (36.5–106.9)	0.093	258.4 (121.7–485.5)	0.103	55.0 (39.5–69.5)	0.005	36.0 (22.0–48.0)	0.023
In the past 6 months	14	7.4 (7.2–7.5)		4.5 (3.0–5.8)		49.0 (30.0–74.3)		195.5 (110.6–304.9)		57.5 (37.0–66.8)		33.0 (21.5–39.0)	
3 months before pregnancy	29	7.5 (7.2–7.7)		3.3 (2.3–4.5)		66.7 (49.9–102.4)		198.0 (157.9–297.5)		54.0 (42.0–70.0)		41.0 (27.4–51.3)	
Use mobile phones to watch videos, play games, and surf the Internet													
Never used	9	7.5 (7.5–7.6)	0.284	3.0 (1.8–8.5)	0.200	80.4 (37.1–118.4)	0.026	241.2 (119.0–502.7)	0.069	63.5 (42.3–71.3)	0.300	33.5 (14.0–36.8)	0.028
Less than 10 min	28	7.4 (7.4–7.8)		4.1 (3.0–5.3)		54.4 (22.9–102.2)		266.3 (87.4–401.0)		53.0 (31.0–72.0)		33.4 (16.3–40.3)	
10–30 min	91	7.2 (7.2–7.5)		5.0 (3.0–6.0)		70.6 (36.9–114.6)		246.0 (145.2–555.0)		61.0 (46.5–72.5)		35.0 (25.0–42.0)	
30 ~ 60 min	115	7.2 (7.2–7.6)		4.0 (2.8–5.6)		63.9 (42.3–103.4)		258.4 (150.0–437.7)		57.0 (44.0–71.0)		38.0 (23.8–50.0)	
60 min and above	222	7.2 (7.2–7.6)		3.9 (2.7–5.0)		64.2 (33.9–103.9)		224.6 (108.1–474.9)		53.0 (35.0–68.0)		36.0 (20.5–49.5)	
Watch TV frequency (on average at least once a week)													
Yes	274	7.5 (7.2–7.6)	0.045	4.0 (2.8–5.6)	0.039	63.5 (36.5–98.9)	0.052	224.6 (116.8–436.9)	0.083	55.0 (37.0–71.0)	0.002	36.0 (21.8–48.0)	0.031
No	191	7.5 (7.2–7.6)		4.0 (2.8–5.3)		65.2 (38.3–121.6)		255.8 (146.3–506.3)		55.0 (41.0–68.0)		35.0 (22.0–47.0)	
Types of TV screens													
CRT	16	7.3 (7.3–7.5)	0.036	4.0 (3.3–6.8)	0.033	61.1 (26.5–106.6)	0.093	179.0 (89.5–731.5)	0.079	61.0 (29.0–70.5)	0.084	34.0 (20.1–42.0)	0.012
Plasma or back head	5	7.2 (7.0–.)		5.5 (2.5–.)		286.5 (10.4–.)		1575.6 (25.9–.)		50.0 (20.0–.)		30.0 (15.0–55.0)	
Liquid crystal	393	7.5 (7.2–7.6)		4.0 (2.7–5.4)		63.5 (36.1–103.0)		234.7 (122.2–433.0)		55.0 (39.0–69.0)		36.0 (22.0–48.0)	
Other	51	7.4 (7.2–7.6)		3.8 (3.0–5.3)		77.0 (41.7–121.3)		334.2 (144.7–606.4)		53.0 (40.5–72.0)		36.0 (22.0–51.0)	
Average TV watching time per day													
Less than 1 h	298	7.5 (7.2–7.6)	0.173	4.0 (2.7–5.3)	0.153	60.3 (34.4–103.1)	0.222	226.2 (111.0–413.6)	0.220	54.0 (39.5–68.0)	0.076	35.0 (22.0–47.9)	0.029
1 ~ 3 h	147	7.4 (7.2–7.6)		4.2 (3.0–6.0)		70.4 (40.3–109.8)		295.6 (132.7–561.2)		58.0 (38.0–72.5)		36.7 (22.0–48.0)	
3 h and above	20	7.4 (7.2–7.5)		3.7 (2.9–4.6)		79.8 (38.8–107.0)		237.0 (127.0–418.7)		56.0 (42.8–79.5)		36.0 (26.5–48.0)	
Computer using per day													
Less than 8 h	401	7.5 (7.2–7.6)*	0.117	4.0 (2.8–5.5)	0.054	64.2 (39.0–107.8)	0.078	262.3 (127.3–459.9)	0.059	57.0 (41.0–71.0)*	0.178	36.0 (23.0–49.0) *	0.033
8 h and above	64	7.3 (7.2–7.5)		4.0 (3.0–5.5)		50.3 (29.5–97.0)		170.4 (97.4–424.9)		41.0 (29.5–55.5)		28.1 (15.5–36.8)	
Frequency of using or exposing to the following pesticides (within 6 months)													
Never	416	7.5 (7.2–7.6)	0.070	4.0 (2.8–5.5)	0.116	63.9 (38.0–103.9)	0.035	236.3 (123.0–459.8)	0.073	55.0 (40.0–69.0)	0.109	35.0 (22.0–47.0)	0.042
Herbicide	18	7.5 (7.3–7.8)		4.3 (2.8–6.5)		65.4 (43.0–99.9)		299.4 (142.0–502.9)		63.5 (55.5–75.3)		39.0 (22.0–56.0)	
Fungicide	21	7.5 (7.3–7.8)		4.0 (2.9–4.7)		63.9 (38.1–106.9)		233.6 (161.0–298.8)		54.0 (31.0–73.0)		40.5 (19.6–51.8)	
Insecticide	10	7.4 (7.2–7.5)		4.5 (3.6–7.0)		57.0 (10.0–154.0)		404.3 (39.3–673.7)		39.0 (17.5–83.0)		30.0 (19.0–47.0)	
Frequently use or contact with the following organic solvents (within 6 months)													
Never	421	7.5 (7.2–7.6)	0.400	4.0 (2.8–5.3)	0.126	63.5 (37.8–103.8)	0.024	233.6 (122.2–433.1)	0.068	55.0 (40.0–69.0)	0.109	35.0 (22.0–48.0)	0.036
Coating	16	7.2 (7.2–7.5)		4.3 (2.2–6.0)		69.0 (33.4–111.3)		371.6 (98.3–664.2)		50.0 (38.0–67.3)		39.1 (20.5–51.3)	
Paint	7	7.5 (7.0–.)		3.5 (3.2–.)		101.1 (41.6–.)		353.8 (133.1–.)		74.0 (35.0–.)		41.0 (33.5–55.9)	
Adhesive	11	7.6 (7.3–7.7)		3.9 (2.0–5.6)		61.1 (21.8–156.6)		297.5 (73.0–498.6)		55.0 (25.0–78.0)		43.3 (19.3–54.5)	
Industrial cleaners	10	7.5 (7.4–8.0)		6.0 (3.8–8.0)		64.2 (52.9–103.9)		513.4 (150.8–716.6)		67.0 (35.0–75.0)		34.0 (17.8–43.8)	
Exposure to vibration													
Yes	62	7.5 (7.2–7.5)	0.140	4.1 (3.1–5.7)	0.095	76.9 (41.2–114.3)	0.178	294.1 (147.6–570.5)	0.316	57.5 (46.0–74.0)	0.250	36.0 (22.0–47.4)	0.031
No	244	7.5 (7.2–7.6)		4.0 (2.7–5.5)		61.5 (37.8–102.9)		227.6 (119.9–430.8)		54.0 (37.0–68.8)		36.0 (21.5–48.5)	
Exposure to noise													
Never	181	7.5 (7.2–7.7)	0.053	4.0 (2.5–5.1)	0.169	63.5 (43.9–107.8)	0.085	246.0 (138.8–426.7)	0.004	57.0 (42.0–69.0)	0.062	36.0 (24.0–50.0)	0.025
Occasionally	251	7.5 (7.2–7.5)		4.0 (3.0–5.7)		66.1 (34.8–104.1)		262.1 (121.7–461.5)		54.0 (36.0–70.0)		35.0 (20.4–47.0)	
Often	33	7.5 (7.3–7.6)		4.1 (3.4–7.5)		50.5 (27.7–100.9)		163.4 (114.3–613.9)		55.0 (45.0–67.0)		36.0 (23.0–43.5)	
Exposure to radiation (within 6 months)													
Never	388	7.5 (7.2–7.6)	0.140	4.0 (2.8–5.5)	0.016	61.2 (35.5–103.8)	0.156	229.5 (116.6–445.8)	0.057	54.5 (37.0–68.3)	0.224	35.0 (22.0–47.4)	0.043
Occasionally	73	7.5 (7.2–7.6)		4.2 (2.9–6.0)		77.5 (45.9–125.3)		270.1 (149.4–511.8)		59.0 (47.0–74.0)		38.6 (26.5–48.0)	
Often (almost every working day)	4	–		–		–		–		–		–	
Nature of occupation													
Furniture manufacturing	7	7.4 (7.3–7.5)	0.341	5.6 (2.7–7.6)	0.412	70.2 (35.7–106.9)	0.617	237.6 (148.1–641.0)	0.704	57.0 (39.8–70.5)	0.251	41.5 (17.0–52.0)	0.037
Electronics manufacturing	31	7.5 (7.2–7.9)		4.0 (2.8–6.0)		54.2 (41.8–80.7)		199.6 (123.9–465.4)		53.0 (35.0–73.0)		32.0 (26.0–45.0)	
Food processing industry	10	7.5 (7.2–7.6)		5.0 (3.4–6.8)		93.7 (34.9–159.8)		505.9 (159.6–945.6)		62.0 (32.0–77.5)		27.0 (22.0–38.0)	
Toy processing industry	14	7.5 (7.4–7.7)		4.8 (2.0–6.8)		82.5 (13.4–176.9)		267.9 (107.0–827.4)		63.0 (41.0–82.0)		33.5 (28.0–42.0)	
Footwear industry	7	7.5 (7.3–7.7)		2.7 (2.2–3.8)		84.9 (51.3–123.7)		334.0 (181.8–416.2)		69.0 (53.0–76.0)		42.0 (35.0–47.5)	
Chemical manufacturing	9	7.5 (7.2–7.7)		3.5 (2.0–7.0)		91.4 (64.2–196.0)		481.3 (150.8–640.1)		56.0 (52.0–68.0)		44.5 (37.0–59.5)	
Taxi or long-distance transportation	3	7.2 (7.2–.)		6.0 (3.3–.)		122.1 (100.8–.)		604.5 (402.9–.)		67.0 (17.0–.)		33.0 (24.0–49.0)	
Other	384	7.5 (7.2–7.6)		4.0 (2.8–5.1)		63.6 (35.7–103.6)		234.2 (115.5–423.4)		55.0 (39.0–69.0)		36.0 (21.0–48.0)	
Standing or lifting heavy objects for long periods at work													
Yes	77	7.5 (7.2–7.6)	0.028	4.1 (2.5–6.5)	0.026	65.6 (41.7–123.7)	0.076	334.2 (137.1–532.8)	0.097	55.0 (41.5–69.0)	0.011	38.0 (20.8–50.0)	0.024
No	388	7.5 (7.2–7.6)		4.0 (2.8–5.3)		63.5 (33.9–101.1)		226.2 (121.9–433.0)		55.0 (39.0–70.0)		35.0 (22.0–47.9)	
Frequent use of microwave or induction cooker (within a year)													
Yes	186	7.5 (7.2–7.7)	0.059	4.0 (2.6–5.4)	0.008	66.1 (41.7–103.7)	0.026	260.4 (134.5–438.8)	0.011	55.0 (41.0–67.8)	0.020	37.0 (23.3–48.0)	0.035
No	279	7.5 (7.2–7.5)		4.0 (3.0–5.5)		61.1 (35.4–107.8)		234.8 (121.8–472.8)		55.5 (36.3–70.8)		34.0 (20.0–48.0)	
Exposure to chemicals at work													
Yes	31	7.5 (7.2–7.7)	0.026	3.6 (2.5–5.3)	0.031	87.1 (54.8–132.9)	0.103	356.7 (142.7–660.6)	0.085	54.0 (43.3–72.0)	0.010	39.0 (31.0–47.5)	0.022
No	434	7.5 (7.2–7.6)		4.0 (3.0–5.5)		62.2 (37.7–103.2)		234.2 (122.1–433.0)		55.0 (39.0–69.0)		35.0 (22.0–48.0)	
Which type of chemical agents are exposed to at work													
Organic solvents such as formaldehyde	454	7.5 (7.2–7.6)	0.069	4.0 (2.8–5.5)	0.270	62.5 (37.4–103.9)	0.196	234.8 (122.0–438.8)	0.233	55.0 (39.0–69.0)	0.125	36.0 (22.0–48.0)	0.028
Carbon disulfide	4	7.5 (7.2–.)		6.0 (4.5–.)		115.0 (64.2–.)		517.6 (513.4–.)		67.0 (21.0–.)		37.1 (25.5–43.1)	
Lead and its compounds	4	7.6 (7.5–.)		3.5 (3.0–.)		67.8 (42.0–.)		245.4 (126.0–.)		61.0 (44.0–.)		38.0 (32.6–38.5)	
Benzene or benzene homologues (toluene, xylene)	3	7.5 (7.2–.)		3.0 (2.0–.)		135.2 (74.3–.)		466.3 (148.3–.)		48.0 (44.0–.)		40.5 (36.0–45.0)	

The value of pH value, semen volume, sperm concentration, sperm count, total motility and progressive motility represent median (25th, 75th percentiles).

*P < 0.05.

### Independent predictors of low semen quality in Binomial logistic regression analysis

**Figure 1:**
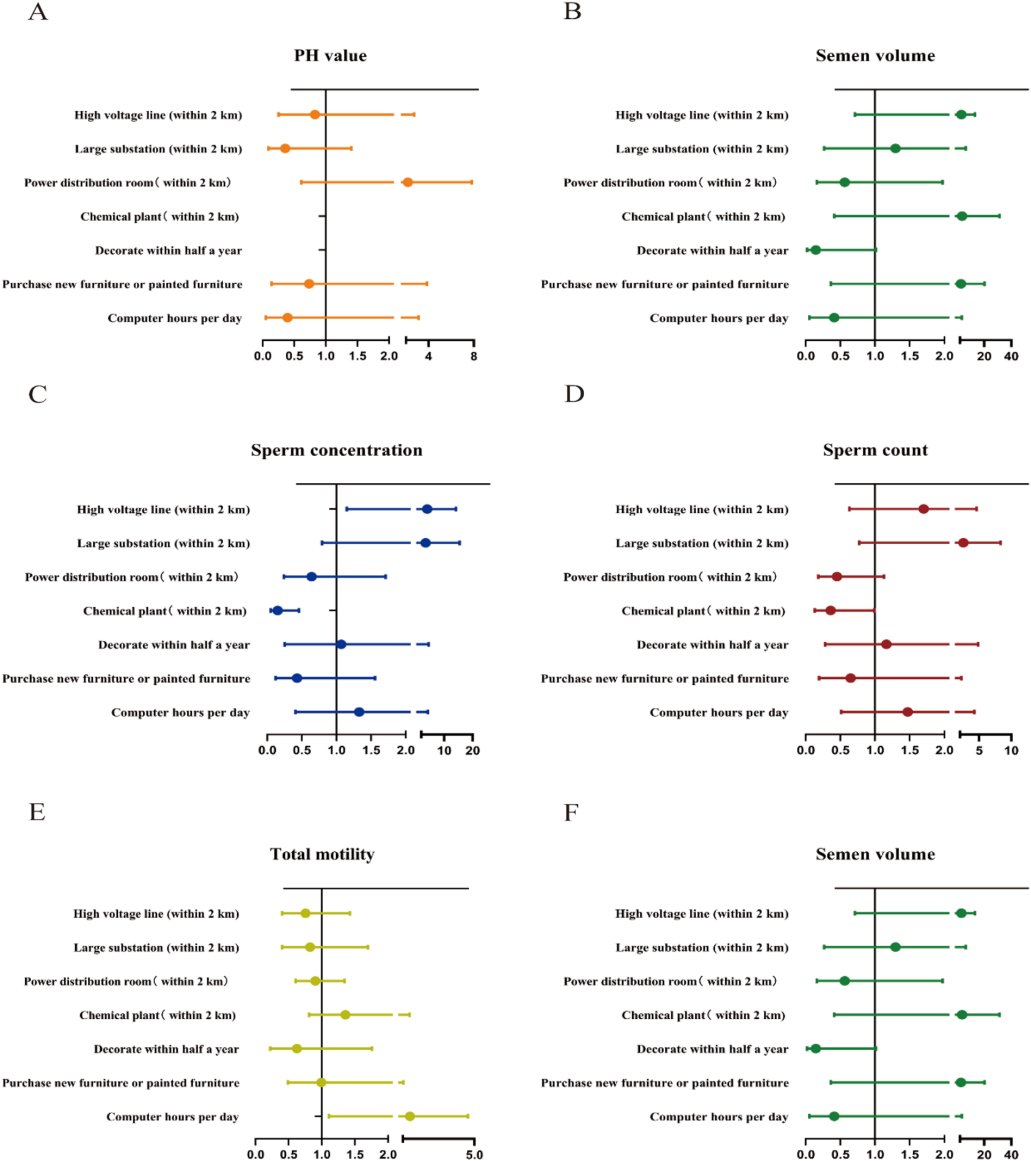
Forest plots show the effect of different occupational and living environmental factors on pH value (**A**), semen volume (**B**), sperm concentration (**C**), sperm count (**D**), total motility (**E**), progressive motility (**F**). Dots represent Adjusted ORs. Error bars indicate 95% CIs.

Table 4 and Fig. 1 shows the binomial logistic analysis results. Abnormal semen quality parameters were defined according to the guidelines of the World Health Organization^15^. After adjusting for potential confounders(age and BMI), our results show that to shorten the time length using the computer within a day is a protective factor to total sperm motility (Adjusted OR 2.29; 95% CI 1.11–4.73;*P* = 0.025; R^2^ = 0.041) And living close to high voltage line is a positive factor for higher sperm concentration (Adjusted OR 4.03; 95% CI 1.15–14.18; *P* = 0.030; R^2^ = 0.048). But living close to a chemical plant is a significant protective factor for higher semen concentration (Adjusted OR 0.15; 95% CI 0.05–0.46; *P* = 0.001; R^2^ = 0.048) and a higher total sperm count (Adjusted OR 0.36; 95% CI 0.13–0.99; *P* = 0.049; R^2^ = 0.026). In addition, after adjusting for confounding factors, the effect of computer use time on sperm progressive motility (%) becomes not significant. (Adjusted OR 1.07; 95% CI 0.57–1.10; *P* = 0.835; R^2^ = 0.038), it seems that this factor is more influenced by BMI or age.

**Table 4:** Binomial regression model to explore the relationship between occupational environmental factors and semen quality.

Semen parameters	Statistical value	High voltage line (within 2 km)	Large substation (within 2 km)	Power distribution room (within 2 km)	Chemical plant (within 2 km)	Decorate within half a year	Purchase new furniture or painted furniture	Computer hours per day
pH value (< 7.2vs ≥ 7.2)	OR (95%CI)	0.77 (0.23–2.57)	0.39 (0.10–1.56)	2.32 (0.63–8.51)	114,783,789.44 (0.00-.)	104,561,701.02 (0.00-.)	0.72 (0.14–3.57)	1.70 (0.44–6.55)
	*P*	0.667	0.184	0.204	0.997	0.998	0.684	0.441
	Adjusted OR (95%CI)	0.83 (0.25–2.73)	0.36 (0.09–1.40)	2.18 (0.61–7.82)	123,422,198.63 (0.00-.)	98,268,263.53 (0.00-.)	0.74 (0.14–3.85)	0.39 (0.05–3.14)
	*P*^*d*^	0.758	0.140	0.231	0.997	0.998	0.718	0.379
	*R2*	0.069						
Semen volume (< 1.5 ml vs ≥ 1.5 ml)	OR (95%CI)	3.65 (0.87–15.42)	1.00 (0.21–4.67)	0.60 (0.18–2.05)	3.48 (0.39–31.28)	0.19 (0.03–1.18)	1.94 (0.27–13.84)	0 (0-.)
	*P*	0.078	0.995	0.417	0.266	0.075	0.508	0.998
	Adjusted OR (95%CI)	3.04 (0.71–13.04)	1.16 (0.24–5.61)	0.56 (0.16–1.98)	3.57 (0.41–31.28)	0.15 (0.02–0.99)	2.69 (0.36–20.18)	0.41 (0.05–3.37)
	*P*^*d*^	0.134	0.857	0.366	0.251	0.049	0.337	0.406
	*R2*	0.039						
Sperm concentration (< 15 × 10^6^/ml vs ≥ 15 × 10^6^/ml)	OR (95%CI)	3.39 (1.02–11.23)	3.11 (0.74–13.13)	0.73 (0.27–1.95)	0.18 (0.06–0.51)	1.10 (0.27–4.48)	0.40 (0.11–1.41)	1.25 (0.37–4.19)
	*P*	0.046	0.123	0.528	0.001	0.897	0.154	0.719
	Adjusted OR (95%CI)	4.03 (1.15–14.18)	3.50 (0.79–15.49)	0.64 (0.24–1.71)	0.15 (0.05–0.46)	1.07 (0.25–4.55)	0.43 (0.12–1.56)	1.32 (0.41–4.33)
	*P*^*d*^	0.030	0.099	0.370	0.001	0.930	0.199	0.642
	*R2*	0.048						
Sperm count (< 39 × 10^6^/ml vs ≥ 39 × 10^6^/ml)	OR (95%CI)	1.66 (0.63–4.43)	2.41 (0.75–7.77)	0.46 (0.18–1.16)	0.38 (0.14–1.03)	1.16 (0.29–4.72)	0.59 (0.17–1.99)	0.79 (0.22–2.89)
	*P*	0.309	0.140	0.101	0.056	0.832	0.390	0.723
	Adjusted OR (95%CI)	1.70 (0.63–4.60)	2.54 (0.77–8.33)	0.45 (0.18–1.13)	0.36 (0.13–0.99)	1.16 (0.28–4.86)	0.64 (0.19–2.22)	1.47 (0.51–4.25)
	*P*^*d*^	0.295	0.125	0.089	0.049	0.837	0.486	0.480
	*R2*	0.026						
Total motility (< 40% vs ≥ 40%)	OR (95%CI)	0.72 (0.38–1.37)	0.83 (0.40–1.73)	1.19 (0.62–2.30)	1.79 (0.78–4.14)	0.61 (0.22–1.70)	1.22 (0.49–3.06)	2.92 (1.40–6.10)
	*P*	0.316	0.623	0.605	0.170	0.341	0.671	0.004
	Adjusted OR (95%CI)	0.75 (0.40–1.43)	0.82 (0.40–1.70)	1.11 (0.57–1.16)	1.72 (0.75–1.94)	0.63 (0.22–1.75)	1.15 (0.46–1.85)	2.29 (1.11–4.73)
	*P*^*d*^	0.385	0.595	0.749	0.201	0.372	0.768	0.025
	*R2*	0.041						
Progressive motility (< 32% vs ≥ 32%)	OR (95%CI)	1.08 (0.63–1.84)	0.75 (0.40–1.41)	1.55 (0.90–2.67)	1.05 (0.54–2.03)	0.67 (0.27–1.64)	1.86 (0.81–4.26)	2.35 (1.19–4.65)
	*P*	0.787	0.372	0.113	0.891	0.380	0.143	0.014
	Adjusted OR (95%CI)	1.15 (0.67–1.96)	0.75 (0.40–1.40)	1.44 (0.84–1.47)	1.08 (0.56–1.08)	0.66 (0.27–1.61)	1.79 (0.79–1.05)	1.07 (0.57–1.10)
	*P*^*d*^	0.621	0.358	0.187	0.828	0.360	0.162	0.835
	*R2*	0.038

## Discussion

### Research status

Twenty-first century to present, experts in the related field had noticed the decreasing trend in human semen quality^28^. There are many different possible causation for the change. It can be due to the unhealthy diet habits, such as alcohol or cigarettes intake^29,30^. But such negative factors can be avoided by accepting doctors’ advice. While organizing a plan for pregnancy, couples need to quit smoking or drinking alcohol as well as carry on healthy diet habits, such as refrain from taking high fat food. By following doctors’ guidance to quit smoking and drinking at least six months before trying to get pregnant, male-partners of couples would always have a better physical condition and semen quality^23,31^, and the chances of successful pregnancy are usually increased^32^. These kind of changes avoid additional expenditure while it usually will lead to a relative remarkable effect. But when it comes to environmental and occupational factors, on account of these factors are always connected to people’s working and living surroundings which are usually much steadier than diet habits, the cost of change is usually much higher. Based on our clinic experience, when we pointed out that one should avoid contacting reproduction toxic substance that existed in their working place^33,34^, they tended to refuse the advice. We didn't regard that they refuse to follow the intervention in an irrational way since it is impossible for an organic chemical worker to completely isolate from chemicals, and the uncertain consequence of quitting their jobs is usually unacceptable. Similarly, to avoid some of the negative factors like noise^12^, and electromagnetic radiation^10,35,36^ around their domicile, they may have to move. In the view of almost all residents, to quit a job or move to a new house just because of giving birth sounds unnecessary, even more so for couples have already raised a child. Under these circumstances, this problem had stuck into a dead loop. The negative factors keep affecting people’s fertility as long as they still exist, but changing their jobs and domicile are remaining unable to afford to most of people.

### Principal findings and comparison with other studies

In this research, we analyzed several factors that may affect semen quality. We have got some results which indicated environmental and occupational factors may affect male’s fertility. Firstly, our result show that living close to power lines and substations are the positive factors for the higher level of sperm count. Besides, living close to a power distribution room may associated to a higher sperm progressive motility. Our data may indicate that electric field energy has a certain effect on semen quality, but the actual effect remains to be further studied and confirmed. Research on effect of electric field to semen quality is relative rare. However, there are also studies that indicate that the electric field effect is related to the decline of semen quality^37,38^, but controversy is existed in academia^39^. These three independent but relevant reports all indicated that electric field may be a beneficial to better sperm quality. But due to most of the power distributions or substations are away from the urban. The population live outside the cities are mainly persons of good economic conditions. which is a well-known fertility related factors which is^40^. Therefore, more experiments should be conducted to verify its effect. Another result shown that living close to a chemical factory may be a negative factor to semen concentration. This result is in accord with other researches, which show that amounts of industrial chemicals will do harm to reproductive system and reduce semen quality^16,41–43^. There are few works had analyzed the association between computer using and semen quality, but related factors (sitting for a long time^44^, electromagnetic wave^45^ and radiation^46^, etc.) had also been reported to be correlated to lower semen quality. It’s still unclear that if using computer or brain work has effect on semen quality, further experiment and researches should be conducted. To figure out the mechanisms of such multi-angle associations are quite challenging but critical issues in the field of public health, especially in the current condition when computers are widely used.

According to our results, we can draw a preliminary conclusion that some of the occupations, and environment factors will affect males’ semen quality. These kinds of factors usually damage human fertility gradually in a cumulative way, because the influence of these factors does not appear as acute diseases. In such condition, people won’t treat the negative factors seriously until they suffer from infertility problems. Fortunately, the negative impact of most factors in our everyday life are reversible. The easiest way is to intervene these factors so that they can avoid their continually damage to our reproduction system. But pregnancy consultation clinics should pay more attention to collect patients’ background information in order to provide personalized a treatment strategy.

The normal quality of semen determines the level of male fertility^47^. Our current results suggest that some environmental and occupational factors may be associated with changes in semen quality. This suggests that changes in environmental and occupational factors may affect male fertility by altering semen quality^48^. By following up the current cohort, we will in the future explore the effects of environmental and occupational factors on prolonged TTP (Time to pregnancy, TTP) due to decreased semen quality^49^.

### Limitation and future researches direction

There were several limitations to our current findings. Firstly, due to semen quality may also be affected differently when exposed to the same occupational or environmental factors^50^. The population of our study is limited to Southern Chinese population, and none of our patients was from other ethnic groups. Secondly, our research only stays at epidemiology level. Thirdly, the existent of confounding factors (such as sleep duration within a day, dietary structure and economic condition, etc.) has interfered part of our results, so in the following research, we will improve our questionnaires to avoid such confounding factors. Fourthly, due to the large number of occupational and environmental factors, we did not include all relevant influencing factors in our analysis, so our current results may not account for the influence of other occupational and environmental factors on semen quality. Fifthly, the effect of dose effects of different factor was not considered in our records yet (such as the length of duration a men lived beside a high voltage line)^51^. In our following research, a modified quantifying will be conducted. Lastly, we only investigated epidemiological risk factors, but what are the specific substances that play a role in each risk factor. Further work should be done to isolate the specific high-risk substances from risk factors, such as specific compounds that may be present around chemical plants that can affect semen quality. In addition, the mechanisms of how high-risk substances affect human sperm quality are still waiting to be explored.

## Conclusion

In summary, our research shown that computer using, living and working surroundings (voltage line, substation and chemical plants, transformer room) and housing decoration are influenced potentially semen quality. However, it is important to note that these findings are based on a limited sample size, and further research with a larger and more diverse population is required to confirm our results. Depending on the characteristics of our population, more different occupational and environmental factors should also be analyzed in our research. Additionally, due to the large number of environmental and occupational factors, we did not include all suspected factors in this study, other factors should be analyzed in future studies. Furthermore, the specific mechanisms through which these risk factors affect semen quality remain unknown, necessitating further investigation.

Overall, our findings highlight the importance of considering the impact of various environmental and occupational factors on semen quality. Continued research in this field will contribute to a better understanding of the potential risks and mechanisms involved, enabling the development of targeted interventions and strategies to support male reproductive health.

## Data Availability

All data generated or analyzed during this study are included in this published article.

## References

[1] Assaysh-Oberg S, Borneskog C, Ternstrom E (2023). Women's experience of infertility & treatment: A silent grief and failed care and support. Sex Reprod. Healthc..

[2] Wang L, Zhu Y, Wang T (2022). Feasibility analysis of incorporating infertility into medical insurance in China. Front. Endocrinol..

[3] Vander Borght M, Wyns C (2018). Fertility and infertility: Definition and epidemiology. Clin. Biochem..

[4] Corsini C, Boeri L, Candela L (2022). Is there a relevant clinical impact in differentiating idiopathic versus unexplained male infertility?. World J. Men Health.

[5] Zarei S, Dehghan SF, Vaziri MH (2022). Assessment of semen quality of taxi drivers exposed to whole body vibration. J. Occup. Med. Toxicol..

[6] Vaziri MH, Sadighi Gilani MA, Kavousi A (2011). The relationship between occupation and semen quality. Int. J. Fertil. Steril..

[7] Irnandi DF, Hinting A, Yudiwati R (2021). DNA fragmentation of sperm in automobile painters. Toxicol. Ind. Health.

[8] Wijesekara GU, Fernando DM, Wijerathna S (2015). Environmental and occupational exposures as a cause of male infertility. Ceylon Med. J..

[9] Gracia CR, Sammel MD, Coutifaris C (2005). Occupational exposures and male infertility. Am. J. Epidemiol..

[10] Adams JA, Galloway TS, Mondal D (2014). Effect of mobile telephones on sperm quality: A systematic review and meta-analysis. Environ. Int..

[11] Abdollahi MB, Dehghan SF, Balochkhaneh FA (2021). Comparison of mice' sperm parameters exposed to some hazardous physical agents. Environ. Anal. Health Toxicol..

[12] Choe SA, Kim S, Im C (2020). Nighttime environmental noise and semen quality: A single fertility center cohort study. PLoS ONE.

[13] Lv MQ, Wang HX, Yang YQ (2022). Semen quality following long-term occupational exposure to formaldehyde in China. JAMA Netw. Open.

[14] Rubes J, Sipek J, Kopecka V (2021). Semen quality and sperm DNA integrity in city policemen exposed to polluted air in an urban industrial agglomeration. Int J. Hyg. Environ. Health.

[15] World Health Organization (2010). WHO Laboratory Manual for the Examination and Processing of Human Semen.

[16] Naha N, Chowdhury AR (2006). Inorganic lead exposure in battery and paint factory: Effect on human sperm structure and functional activity. J UOEH..

[17] Jung A, Schuppe HC (2007). Influence of genital heat stress on semen quality in humans. Andrologia.

[18] Gill K, Jakubik J, Kups M (2019). The impact of sedentary work on sperm nuclear DNA integrity. Folia Histochem. Cytobiol..

[19] Jurewicz J, Radwan M, Sobala W (2014). Effects of occupational exposure: Is there a link between exposure based on an occupational questionnaire and semen quality?. Syst. Biol. Reprod. Med..

[20] Brunoni AR, Valiengo L, Baccaro A (2013). The sertraline vs. electrical current therapy for treating depression clinical study: Results from a factorial, randomized, controlled trial. JAMA Psychiatry.

[21] Glazer CH, Li S, Zhang CA (2019). Racial and sociodemographic differences of semen parameters among US men undergoing a semen analysis. Urology.

[22] Bibi R, Jahan S, Afsar T (2022). The influence of paternal overweight on sperm chromatin integrity, fertilization rate and pregnancy outcome among males attending fertility clinic for IVF/ICSI treatment. BMC Pregnancy Childbirth.

[23] Kulaksiz D, Toprak T, Tokat E (2022). Sperm concentration and semen volume increase after smoking cessation in infertile men. Int. J. Impot. Res..

[24] Amor H, Hammadeh ME, Mohd I (2022). Impact of heavy alcohol consumption and cigarette smoking on sperm DNA integrity. Andrologia.

[25] Petrella F, Lusignan MF, Gabriel MS (2022). Impact of age and fertility status on the consistency of repeat measurements of sperm dna damage: A single-center, prospective, dual visit study. Urology.

[26] Lakens D (2013). Calculating and reporting effect sizes to facilitate cumulative science: A practical primer for t-tests and ANOVAs. Front. Psychol..

[27] Mai, M. *et al*. *Association of Environment and Occupations Factors With Semen Quality in Male Partners of Couples Trying to Conceive, 02 March 2022, PREPRINT (Version 1) available at Research Square* [10.21203/rs.3.rs-1391533/v1]

[28] Virtanen HE, Jorgensen N, Toppari J (2017). Semen quality in the 21(st) century. Nat. Rev. Urol..

[29] Tang Q, Pan F, Wu X (2019). Semen quality and cigarette smoking in a cohort of healthy fertile men. Environ. Epidemiol..

[30] Suliga E, Gluszek S (2020). The relationship between diet, energy balance and fertility in men. Int. J. Vitam. Nutr. Res..

[31] Finelli R, Mottola F, Agarwal A (2021). Impact of alcohol consumption on male fertility potential: A narrative review. Int. J. Environ. Res. Public Health.

[32] Tempest N, France-Ratcliffe M, Al-Lamee H (2022). Habitual physical activity levels in women attending the one stop infertility clinic: A prospective cross-sectional observational study. Reprod. Fertil..

[33] Ribeiro IM, Viana AGA, Carvalho RPR (2022). Could metal exposure affect sperm parameters of domestic ruminants? A meta-analysis. Anim. Reprod Sci..

[34] Louis GM, Chen Z, Schisterman EF (2015). Perfluorochemicals and human semen quality: The LIFE study. Environ. Health Perspect..

[35] Sterling L, Harris LR, Carroll K (2022). The effects of wireless devices on male reproductive health: A literature overview. Rev. Int. Androl..

[36] Hagras AM, Toraih EA, Fawzy MS (2016). Mobile phones electromagnetic radiation and NAD(+)-dependent isocitrate dehydrogenase as a mitochondrial marker in asthenozoospermia. Biochim. Open.

[37] Houston BJ, Nixon B, King BV (2016). The effects of radiofrequency electromagnetic radiation on sperm function. Reproduction.

[38] International Commission on Non-Ionizing Radiation P (2020). Guidelines for limiting exposure to electromagnetic fields (100 kHz to 300 GHz). Health Phys..

[39] Lewis RC, Hauser R, Maynard AD (2016). Exposure to power-frequency magnetic fields and the risk of infertility and adverse pregnancy outcomes: Update on the human evidence and recommendations for future study designs. J. Toxicol. Environ. Health B.

[40] Muhamad S, Sengupta P, Ramli R (2019). Sociodemographic factors associated with semen quality among Malaysian men attending fertility clinic. Andrologia.

[41] Bernard A (2022). Dermal exposure to hazardous chemicals in baby diapers: A re-evaluation of the quantitative health risk assessment conducted by the French agency for food, environmental and occupational health and Safety (ANSES). Int. J. Environ. Res. Public Health.

[42] Calvert L, Green MP, De Iuliis GN (2021). Assessment of the emerging threat posed by perfluoroalkyl and polyfluoroalkyl substances to male reproduction in humans. Front. Endocrinol..

[43] Tian T (2018). Association of bisphenol A exposure with LINE-1 hydroxymethylation in human semen. Int. J. Environ. Res. Public Health.

[44] Koskelo R, Zaproudina N, Vuorikari K (2005). High scrotal temperatures and chairs in the pathophysiology of poor semen quality. Pathophysiology.

[45] Kamali K, Atarod M, Sarhadi S (2017). Effects of electromagnetic waves emitted from 3G+wi-fi modems on human semen analysis. Urologia.

[46] Wdowiak A, Stec M, Raczkiewicz D (2020). Background ionizing radiation and semen parameters of men with reproductive problems. Ann. Agric. Environ. Med..

[47] Buck Louis GM, Sundaram R, Schisterman EF (2014). Semen quality and time to pregnancy: The Longitudinal Investigation of Fertility and the Environment Study. Fertil. Steril..

[48] Wu W, Chen Y, Cheng Y (2022). Association between ambient particulate matter exposure and semen quality in fertile men. Environ. Health.

[49] Snijder CA, te Velde E, Roeleveld N (2012). Occupational exposure to chemical substances and time to pregnancy: A systematic review. Hum. Reprod. Update.

[50] Khandwala YS, Zhang CA, Li S (2017). Racial variation in semen quality at fertility evaluation. Urology.

[51] Ramos-Flores A, Camacho-Hernandez I, Sierra-Santoyo A (2021). Temephos decreases sperm quality and fertilization rate and is metabolized in rat reproductive tissues at low-dose exposure. Toxicol. Sci..

